# Utility of 1,2‐o‐dilauryl‐rac‐glycero glutaric acid‐(6′‐methylresorufin)‐ester‐lipase for monitoring dogs with chronic pancreatitis

**DOI:** 10.1111/jvim.16638

**Published:** 2023-02-13

**Authors:** Sharon Kuzi, Dana Adlersberg, Itamar Aroch, Gilad Segev

**Affiliations:** ^1^ Department of Small Animals Internal Medicine Hebrew University Veterinary Teaching Hospital and Koret School of Veterinary Medicine, Hebrew University of Jerusalem Rehovot 761001 Israel

**Keywords:** abdominal pain, acute on chronic pancreatitis, canine, DGGR‐lipase

## Abstract

**Background:**

The utility of 1,2‐o‐dilauryl‐rac‐glycero glutaric acid‐(6′‐methylresorufin)‐ester‐(DGGR)‐lipase activity (DLA) in monitoring clinical progression of chronic pancreatitis (CP) in dogs is unknown.

**Objective:**

To examine the association of DLA with clinical signs of CP, as assessed by a CP clinical severity score (CPCSS).

**Animals:** Twenty‐four dogs.

**Methods:**

This is a retrospective study. Chronic pancreatitis was diagnosed based on clinical signs and DLA > 250 U/L and monitored using CPCSS and DLA.

**Results:**

The study included 134 visits (median, 10 visits/dog; range, 2‐11). Mild‐moderate (CPCSS, 0‐3) and severe (CPCSS, ≥4) disease were documented in 94 (70%) and 40 (30%) visits, respectively. In emergency visits (n = 44; 33%) CPCSS (median, 5; range, 0‐15) and DLA (median, 534 U/L; range, 63‐7133) were higher (*P* < .001 and *P* = .003, respectively) than in scheduled ones (n = 90; 67%; median, 1; range, 0‐6 and median, 384 U/L; range, 49‐3747, respectively). DGGR‐lipase activity was associated (*P* = .009) with the CPCSS, with a lower activity documented in mild‐moderate CPCSS (median 391 U/L; range, 49‐3747), compared to severe score (median, 558 U/L; range, 63‐7133). DGGR‐lipase activity was significantly, but weakly, correlated with CPSS (*r* = 0.233, *P* = .007). DGGR‐lipase activity inefficiently discriminated mild‐moderate vs severe CP (area under the receiver operator characteristics curve, 0.64; 95% confidence interval, 0.53‐0.75; *P* = .012), with DLA cutoff of 428 U/L corresponding to sensitivity of 65% and specificity of 63%.

**Conclusions and Clinical Importance:**

Increased DLA is associated with emergency revisits in dogs with CP, possibly reflecting acute flare‐ups. DGGR‐lipase activity was associated with the CPCSS over the follow‐ups but could not differentiate disease severity.

**Abbreviations**
A/CP: acute on chronic pancreatitis
AP: acute pancreatitis
CIBDAI: canine inflammatory bowel disease activity index
CIE: chronic inflammatory enteropathy
CKD: chronic kidney disease
CP: chronic pancreatitis
CPCSS: chronic pancreatitis clinical severity score
cPLI: canine pancreatic lipase immunoreactivity
DGGR‐lipase: 1,2‐o‐dilauryl‐rac‐glycero glutaric acid‐(6′‐methylresorufin) ester‐lipase
RI: reference interval

## 1 INTRODUCTION

Chronic pancreatitis (CP) is continuous pancreatic inflammation characterized by irreversible histopathological lesions and possibly leading to pancreatic exocrine and endocrine function loss.^1^, ^2^ Chronic pancreatitis presents a diagnostic challenge in dogs, because of its variable nonspecific clinical presentation (including vomiting, inappetence, and abdominal pain, ranging from subclinical to debilitating disease, and nonspecific laboratory findings), which could be attributed to comorbidities.^3^, ^4^, ^5^

Necropsy‐based, histological studies suggest that CP is common in dogs, with a prevalence of 34%‐64% in all necropsied dogs.^1^, ^6^ However, some necropsy‐based studies do not distinguish between different histopathologic forms of CP, and do not correlate histologic abnormalities with clinical findings.^1^, ^7^, ^8^, ^9^, ^10^ Consequently, the clinical importance of postmortem findings of pancreatic inflammation and fibrosis are mostly unknown.^1^, ^7^, ^8^, ^9^, ^10^ Obtaining pancreatic biopsies antemortem is invasive. Moreover, biopsies might be diagnostically insensitive to detect mild or early CP, because of patchy lesion distribution and limited tissue sample size.^1^, ^6^ Considering the associated morbidity and the limited impact on clinical decision‐making, pancreatic biopsies are mostly not acquired in the clinical setting.^2^

There is no validated system for diagnosing CP in dogs. Similar to human medicine, noninvasive diagnosis of CP relies on presence of appropriate clinical signs, diagnostic imaging and pancreatic‐specific laboratory test findings, and vigorously ruling out concurrent diseases.^2^ Common clinical signs of dogs with histologically confirmed CP included lethargy (80%), decreased appetite (70%), vomiting (63%), diarrhea (36%), and abdominal pain (27%).^4^, ^5^ Aside from abdominal pain, these clinical characteristics comprise the canine inflammatory bowel disease activity index (CIBDAI),^11^ which is useful for monitoring and prognosticating chronic inflammatory enteropathies (CIEs).^12^, ^13^ Recently, a modified CIBDAI‐based canine activity index showed excellent prognostic accuracy in dogs with acute pancreatitis (AP).^14^ Therefore, similar clinical severity index scoring systems might serve in monitoring and prognosticating CP in dogs. Nevertheless, such an index has not been evaluated.

Pancreatic lipases, such as 1,2‐o‐dilauryl‐rac‐glycero glutaric acid‐(6′‐methylresorufin) ester‐(DGGR)‐lipase, or serum canine pancreatic lipase immunoreactivity (cPLI), are useful in the diagnosis of AP.^15^, ^16^, ^17^, ^18^ These were evaluated in only small cohorts of dogs with CP, with unsatisfactory sensitivity of 42%‐67%.^4^, ^15^ Combining increased serum cPLI concentration and presence of pancreatic sonographic abnormalities has diagnostic sensitivity <60% for CP.^5^ Possibly, pancreatic tissue loss, alongside minimal tissue edema and inflammation, contribute to the low diagnostic sensitivity of pancreatic lipase assays and sonography.^2^ Nevertheless, both cPLI and DGGR‐lipase were 100% diagnostically specific in 8 dogs with histologically confirmed CP.^15^ Additionally, higher serum pancreatic lipase activities occur more frequently in nonsurviving dogs with AP and cPLI concentrations correlated with an AP clinical severity score, suggesting that both are disease severity markers.^14^, ^19^, ^20^ Similar data of the utility of pancreatic lipases in monitoring and prognosticating dogs with CP are unavailable.

In addition to pancreatic lipase essays, C‐reactive protein (CRP) was significantly and positively correlated with the clinical severity of AP in dogs, as well as with serum cPLI concentration.^14^, ^21^ C‐reactive protein is a readily available and a commonly used acute phase protein for assessing disease severity and monitoring response to treatment in various chronic inflammatory diseases in dogs (eg, polyarthritis and CIEs),^11^, ^22^, ^23^ but the clinical utility of CRP in CP in dogs, or its correlations with DGGR‐lipase activity and clinical signs were not investigated.

The main aim of this retrospective study was to examine the association of a clinical severity scoring index, comprised of the CIBDAI criteria, alongside abdominal pain, with DGGR‐lipase activity, and to assess their utility in monitoring dogs diagnosed with CP as the sole or major disease. An additional aim was to assess the correlations between CRP and either the clinical severity scoring index score, or DGGR‐lipase activity, to assess its utility as an additional marker of disease severity, alongside evidence of systemic inflammation, as manifested by CRP, in dogs with CP. The study hypothesis was that serum DGGR‐lipase activity will be positively associated with the CPCSS, and will thus be a useful marker in monitoring dogs with CP, similarly to its diagnostic utility in dogs with AP.^14^, ^18^, ^19^, ^20^

## 2 MATERIALS AND METHODS

### 2.1 Selection of dogs, definitions, and data collection

This was a retrospective study, including dogs diagnosed with CP in a referral veterinary teaching hospital (VTH) between 2017 and 2021. Chronic pancreatitis was diagnosed based on the combined presence of compatible chronic or intermittent (≥3 weeks duration) gastro‐intestinal clinical signs, including ≥2 of the following: hyporexia, vomiting, diarrhea, abdominal pain,^14^ and ≥1 documentation of DGGR‐lipase activity >250 U/L (reference interval [RI], 5‐107 U/L) at any point during the follow‐up period. 1,2‐o‐dilauryl‐rac‐glycero glutaric acid‐(6′‐methylresorufin) ester‐lipase activity was measured by a colorimetric lipase assay (LIPC, Roche, Mannheim, Germany) using an autoanalyzer (Cobas 6000; at 37°C) as previously reported.^17^ A DGGR‐lipase activity cutoff of >250 U/L was chosen based on a previous study, where this same assay was used, that reported an equivocal range of 109‐216 U/L.^17^ Thus, the chosen cutoff in the present study is conservative and considered more specific for pancreatitis. A similar serum DGGR‐lipase activity cutoff (>245 U/L), albeit using a different DGGR‐lipase assay, was considered to have 100% specificity for AP or CP in another previous study.^15^ Abdominal ultrasonography was performed in all dogs, although presence of sonographic changes indicative of CP (ie, pancreatic mixed‐echogenicity or hyper‐echogenicity)^24^, ^25^ was not a prerequisite inclusion criterion. Only dogs followed for ≥2 revisits scheduled for monitoring and management of CP, with available DGGR‐lipase activities were considered.

The study included dogs with and without concurrent diseases, fed various diets, and treated by different treatment regimens, as these represent the complex clinical actuality, the therapeutic and monitoring challenges in canine CP. Dogs were included with concomitant diseases only if these were deemed secondary processes, with minimal or no contribution to the clinical signs. For example, dogs with chronic kidney disease (CKD) with serum creatinine concentration ≤2.0 mg/dL during all revisits were included, as CKD was deemed subclinical, with minimal or no contribution to the relevant clinical signs. Dogs with diabetes mellitus (DM) and hyperadrenocorticism were included only if deemed stable, with no diabetic ketoacidosis or iatrogenic hypocortisolemia during follow‐up. Dogs treated for lymphoma and multiple myeloma and in complete remission were also included, if CP was considered the main disease process responsible for clinical signs.

Dogs were excluded if comorbidities potentially had a major contribution to the clinical signs, or if such comorbidities could not be ruled out based on physical examination, CBC, serum chemistry, and abdominal ultrasound (performed in all dogs included upon diagnosis of CP). Efforts were made to exclude dogs with concomitant CIEs and hepatobiliary diseases. The classical presentation of CP is described as waxing and waning, mostly low‐grade, anorexia, and gastrointestinal signs.^2^ Therefore, dogs were excluded if pre‐existing CIEs were suspected to cause persistent, rather than intermittent, gastrointestinal disease, or with presence of sonographic findings of increased intestinal mucosal echogenicity and enlarged mesenteric lymph nodes,^26^ abnormal intestinal histological findings, or abnormally low intestinal absorptive function. Unless attributed to DM and hyperadrenocorticism, or to corticosteroid therapy, dogs with abnormally high alanine aminotransferase, alkaline phosphatase, and gamma‐glutamyl transferase activity above 1.5‐fold their upper RIs were excluded, unless available liver histology ruled out primary hepatobiliary diseases. Visits in which DGGR‐lipase or clinical signs were not fully recorded, were excluded.

Historical AP (before inclusion) or acute flare‐up of chronic pancreatitis (A/CP), or A/CP episodes during the study period, were recorded when dogs were presented to the emergency service with acute historical clinical signs or acute clinical deterioration during the follow‐up period, with compatible clinical signs of anorexia, frequent vomiting and marked lethargy,^14^ and sonographic evidence of pancreatomegaly, pancreatic hypoechogenicity, peri‐pancreatic hyperechoic mesentery, and peri‐pancreatic abdominal free fluid.^24^, ^25^

After the diagnosis of CP and evaluation of comorbidities, data collected on each visit included the clinical signs and DGGR‐lipase activity. Serum CRP concentration (Canine CRP, Randox Laboratories LTD, Crumlin, UK)^27^, ^28^ was recorded when available. Additionally, data pertaining to the severity of CP at each visit were recorded, including the type of revisit (ie, a scheduled elective follow‐up visits or unscheduled emergency revisits suggestive of A/CP), and administration of supportive therapy (eg, antiemetics, analgesics). Revisits were scheduled at the attending internal‐medicine clinicians' discretion, to assess the clinical response to treatment changes, and to modify therapeutic interventions when warranted, as well as to monitor DGGR‐lipase activity. Surviving dogs were defined as those surviving during the 3‐month period after the last recorded visit.

### 2.2 Chronic pancreatitis clinical severity score

A CP clinical severity score (CPCSS) system was utilized herein, based on presence and severity of the clinical signs comprising the CIBDAI (ie, attitude, appetite, vomiting, feces consistency, feces frequency, and weight loss),^11^ with addition of abdominal pain (Table 1), based on the history provided by dogs' owners, and physical examination findings in each visit, as recorded in the medical records. Each clinical sign was assigned a score as follows: 0 (normal), 1 (mild), 2 (moderate), and 3 (severe).^11^ Abdominal pain was scored as present (score = 1) or absent (score = 0). Vomiting and fecal frequency and consistency were scored based on the history of the 7 days before each visit, whereas the attitude, appetite, and presence of abdominal pain were scored based on data recorded in the physical examination on each visit. Changes in body weight were compared to the body weight recorded in the previous visit. The scores of each clinical sign were assigned by a single primary investigator. The final CPCSS was comprised of the sum of individual scores, and could range between 0 and 19 points. The CPCSS was recorded on each visit, and was categorized as follows: 0‐3, mild‐moderate; ≥4, severe; based on an association (*P* < .001) between occurrence of an emergency visit and CPCSS ≥4.

**TABLE 1:** Canine chronic pancreatitis clinical severity scoring system, based on chronic inflammatory bowel disease activity index and the modified canine acute pancreatitis activity index.^11^, ^14^

Criterion	No clinical signs	Mild clinical signs	Moderate clinical signs	Marked clinical signs
	Score 0	Score 1	Score 2	Score 3
Attitude/activity	Normal	Slightly decreased	Moderately decreased	Markedly decreased
Appetite	Normal	Slightly decreased	Moderately decreased	Markedly decreased
Vomiting	None	Mild (1/week)	Moderate (2‐3/week)	Severe (>3/week)
Feces consistency	Normal	Slightly soft feces	Very soft feces	Watery diarrhea
Feces frequency	Normal	Slightly increased (2‐3 times a day) or fecal blood, mucus, or both	Moderately increased (4‐5 times a day)	Markedly increased (>5 times a day)
Weight loss	None	Mild (<5%)	Moderate (5%‐10%)	Marked (>10%)
Abdominal pain	None	Abdominal wall resistance,^a^ or the dog resists abdominal palpation, or presence of other signs of pain elicited upon abdominal palpation; the dog ambulates slowly, or is reluctant to ambulate or lie down; occasional praying position noted at home; occasional or persistent unusual vocalization		

^a^ Abdominal pain was recorded as absent (0) or present (1), regardless of severity.

### 2.3 Statistical analysis

Normality was assessed using the Shapiro‐Wilk's test. The Mann‐Whitney's test was used to compare continuous variables (eg, DGGR‐lipase activities) between groups. Associations between categorical parameters (eg, type of visit) were evaluated using the Chi‐square or Fisher's exact tests, as appropriate. The CPCSS was then divided into a severe and a mild‐moderate clinical disease severity categories, based on significant differences in frequency of emergency visits in each CPCSS level category. Associations between DGGR‐lipase activity and severity of CP (ie, CPCSS) during the follow‐up period were examined using generalized linear equations. Receiver operator characteristics analysis of clustered data, with its area under the curve (ROC AUC) and its 95% confidence interval (95% CI), was performed to assess the diagnostic accuracy of serum DGGR‐lipase activity in classifying the clinical severity, as reflected by the CPCSS. The clinical score was categorized to mild‐moderate (CPCSS, 0‐3) vs severe (CPCSS ≥4) CP. The optimal serum DGGR‐lipase activity cutoff values, with their corresponding sensitivity and specificity, were those associated with the least number of misclassifications, chosen using the Youden index. Associations between continuous variables were examined using the Spearman's correlation test. All tests were 2‐tailed, and *P* < .05 was considered significant in all. Statistical analyses were performed using statistical software packages (SPSS 28.0.1.0, IBM, Armonk, New York; STATA 15, Stata Corp., College Station, Texas).

## 3 RESULTS

### 3.1 Signalment, history, concurrent diseases, and treatments

The study included 24 dogs with CP (neutered females, 13; 54%; males, 11; 46%; neutered, 9; 82%), of the following breeds: mixed breed (16 dogs; 67%), toy poodle (2; 8%), and Belgian Malinois, Maltese, Yorkshire terrier, Shih tzu, miniature schnauzer, and Weimaraner (1 each; 4%). The median age was 12.5 years (range, 7.5‐17.0), and the median body weight was 9.4 kg (range, 2.4‐45.7).

In 20 dogs (83%), at least 1 episode of AP or A/CP was documented before inclusion in this study (1 episode, 7 dogs [25%]; 2 episodes, 6 dogs [25%]; 3 episodes, 1 dog [4%]; 4 episodes, 2 dogs [8%]). The median duration of historical CP‐associated clinical signs, before the first recorded visit, was 55 days (range, 21‐270 days). Sonographic pancreatic abnormalities suggestive of CP were noted in 9 dogs (37%) upon their enrollment. The results of the CBC and serum chemistry upon diagnosis of CP are presented in Tables S1 and S2.

One concurrent chronic disease was diagnosed in 16 dogs (67%), including 6 dogs (25%) with stable (ie, no change during the follow‐up period) CKD, of which 1 dog was classified as International Renal Interest Society (IRIS) Stage‐1, and 5 dogs as IRIS Stage‐2,^29^ 4 dogs (17%) with DM, 2 dogs (8%) with neoplasia in remission (lymphoma and multiple myeloma), and 1 dog (4%) each of hyperadrenocorticism, hypothyroidism, mild portal lymphocytic hepatitis, and familial hypertriglyceridemia of Miniature Schnauzer. Endocrinopathies were treated with trilostane and levothyroxine, respectively, with no treatment changes made during the follow‐up period. In 3 additional dogs (12%), 2 concurrent chronic diseases were diagnosed, including DM with IRIS stage‐2 CKD (2 dogs; 8%) and DM with hyperadrenocorticism (1 dog; 4%).

The study included 134 follow‐up visits overall (median number of visits per dog, 10; range, 2‐11), of which 90 (67%) were scheduled elective visits, and 44 (33%) were emergency visits (of which in 30 [68%], dogs were hospitalized). The median overall follow‐up period (from first to last visit) was 163 days (range, 14‐1247). The median frequency of visits was every 17 days (range, 3‐58), while the median between‐visit interval was 31 days (range, 3‐63), and the median interval between visits when DGGR‐lipase activity was >250 U/L was 30.5 days (range, 5‐619). All dogs survived during the follow‐up period.

Low‐fat commercial prescription dry diets were exclusively administered in 116 visits (87%), including Hill's Prescription Diet i/d low fat (Etten‐Leur, the Netherlands), Purina Pro Plan Veterinary Diets Overweight Management Canine Formula (Portogruaro, Italy), and Royal Canin Gastrointestinal low‐fat (Aimargues, France), whereas in 18 (13%), such diets were combined with low‐fat homemade diet (fat <25% metabolizable energy). Supportive symptomatic therapy was recorded in 115 visits (86%), including antiemetics (84 visits [63%]; maropitant‐citrate [cerenia, Pfizer PGM, France], 74 visits [55%]; metoclopramide [pramin, RAFA Laboratories LTD, Jerusalem, Israel], 12 visits [9%]), omeprazole (omepradex, [Dexel Pharma, Or Akiva, Israel], 40 visits; 30%), appetite stimulant (mirtazapine [mirtazapine, Teva Pharmaceuticals Industries, Tel Aviv, Israel] in 74 visits; 55%), and analgesia (maropitant citrate, 74 visits; 55%). Five dogs (21%; 53 visits, 40%) were administered immunosuppressives (prednisone, generic [Rekah Pharmaceutical Industries, Holon, Israel], 2 dogs, 8%; leflunomide [arava, Sanofi Winthrop Industrie, Paris, France], 3 dogs; 12%).

### 3.2 Chronic pancreatitis clinical severity score indices and serum DGGR‐lipase activity

**FIGURE 1:**
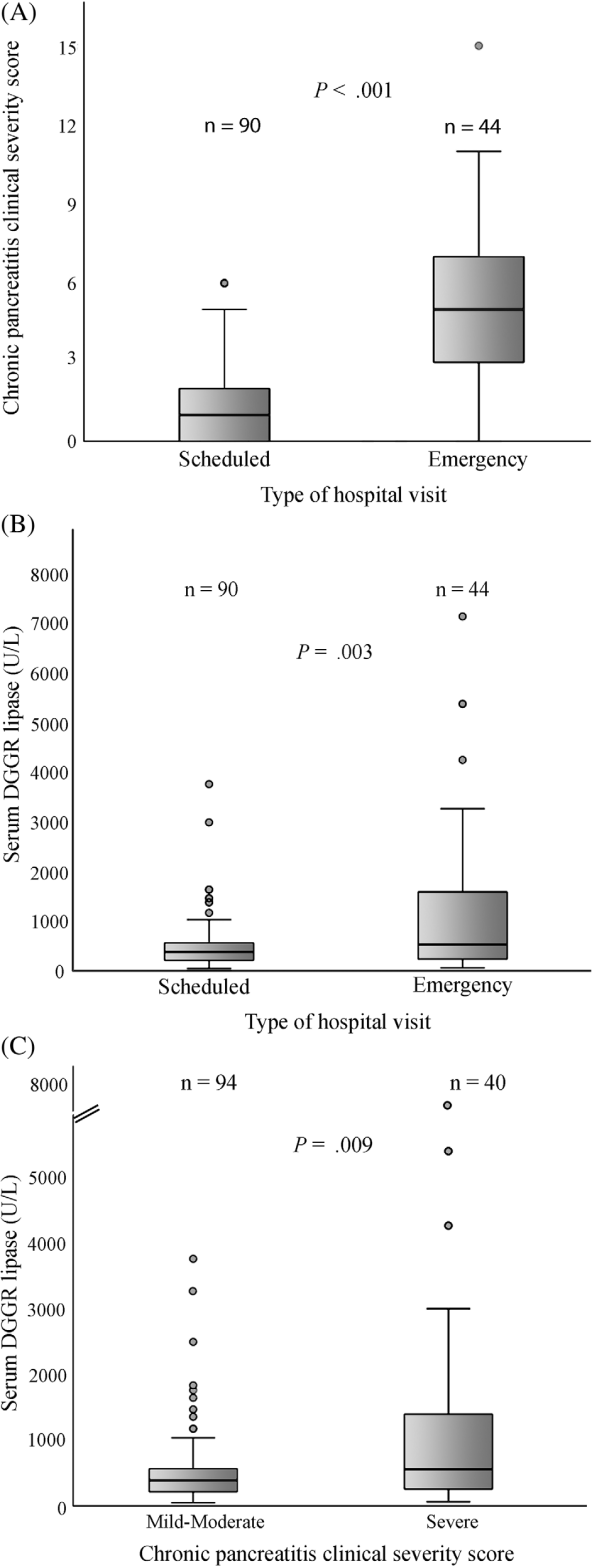
Data are presented as boxes and whiskers. The horizontal line within the box represents the median, the box represents the 25th to 75th percentile. The whiskers represent the ranges, extending up to 1.5 the interquartile range. Outliers are represented with circles. (A) Clinical score of scheduled vs emergency visits. (B) 1,2‐o‐dilauryl‐rac‐glycero glutaric acid‐(6′‐methylresorufin) ester (DGGR)‐lipase activity of scheduled follow‐ups compared to unscheduled emergency revisits. (C) DGGR‐lipase activity in different levels of chronic pancreatitis clinical severity (CPCS) score, as follows: mild‐moderate (CPCS score, 0‐3) and severe (CPCS score, ≥4).

The scores of each individual clinical sign that were assigned during the follow‐up period, which collectively formed the CPCSS, are summarized in Table 2. Clinical signs recorded were hyporexia (59 revisits; 44%), weight loss (58 revisits, 43%), attitude changes (44 revisits, 29%), vomiting (32 revisits, 24%), abdominal pain (29 revisits, 22%), abnormal fecal consistency (28 revisits, 21%), and increased defecation frequency (6 revisits, 4%). The associations between serum DGGR‐lipase activity and each clinical sign are presented in Table S3. The CPCSS was higher (*P* < .001) in unscheduled emergency visits (median, 5; range, 0‐15) compared to elective visits (median, 1; range, 0‐6; Figure 1A). Serum DGGR‐lipase activity was also higher (*P* = .003) on emergency visits (median, 534 U/L; range, 63‐7133) compared to elective visits (median, 384 U/L; range, 49‐3747; Figure 1B).

**TABLE 2:** The frequency and assigned scores of individual clinical signs comprising the chronic pancreatitis clinical severity scoring system in 134 visits of 24 dogs diagnosed with chronic pancreatitis.

Score^a^	Attitude	Appetite	Abdominal pain	Vomiting	Body weight	Defecation frequency	Feces consistency
	n (%)	n (%)	n (%)	n (%)	n (%)	n (%)	n (%)
0	93 (71)	75 (56)	105 (78)	102 (76)	76 (57)	128 (96)	106 (79)
1	34 (26)	39 (29)	29 (22)	21 (16)	43 (32)	2 (1)	16 (12)
2	7 (3)	7 (5)		9 (7)	12 (9)	3 (2)	7 (5)
3	0 (0)	13 (10)		2 (1)	3 (2)	1 (1)	5 (4)

*Note*: n, number of revisits in which specific scores were assigned to each clinical sign.

^a^ See text for details of the scoring of each clinical sign.

Based on the significant association between frequency of emergency unscheduled revisits and the CPCSS level, the CPCSS scores were categorized as mild‐moderate (94/134 visits; 70%, including 81/90 planned visits; 90%) and severe (40/134 visits; 30%, including 31/44 emergency visits; 70%, *P* < .001) CP. Serum DGGR‐lipase activity differed (*P* = .009) between the CPCSS categories, with lower activity measured in mild‐moderate CP (CPCSS 0‐3; median, 391 U/L; range, 49‐3747), compared with severe CP (CPCSS score ≥4; median, 558 U/L; range 63‐7133; Figure 1C).

**FIGURE 2:**
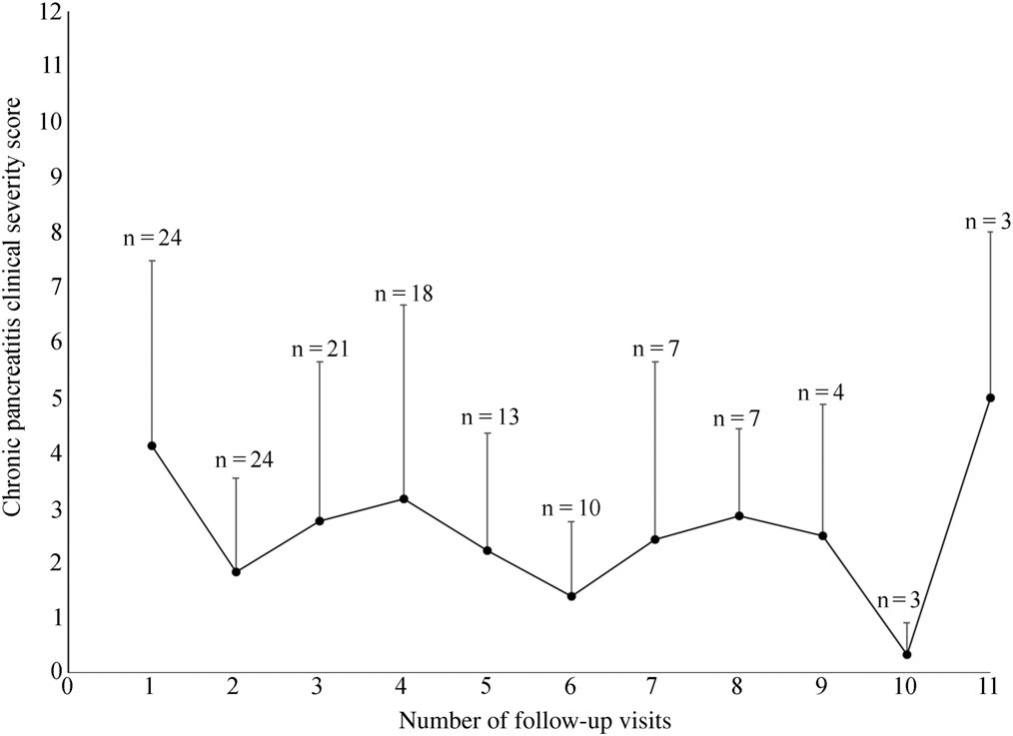
Chronic pancreatitis clinical severity (CPCS) score through 11 follow‐up visits from diagnosis of 24 dogs with chronic pancreatitis presented to the hospital. In each visit, the dot represents the mean CPCS score, and the whisker represents the SD. “n” is the number of dogs evaluated in each re‐visit.

**FIGURE 3:**
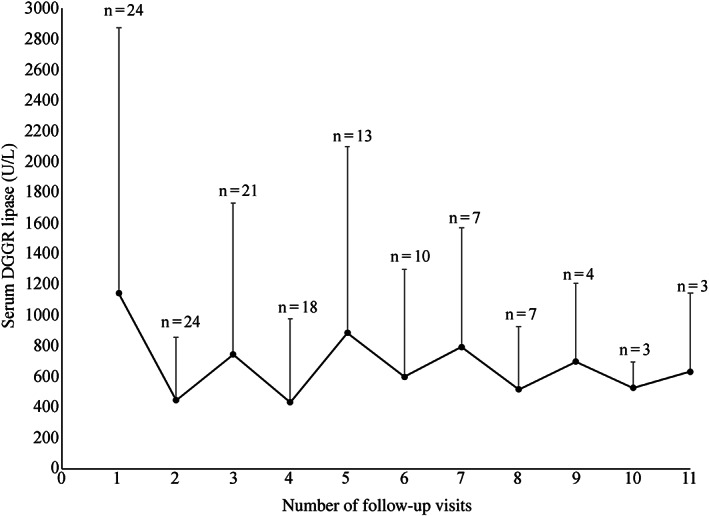
Mean and SD of 1,2‐o‐dilauryl‐rac‐glycero glutaric acid‐(6′‐methylresorufin) ester (DGGR)‐lipase activity through 11 follow‐up visits of 24 dogs with chronic pancreatitis presented to the hospital. In each visit, the dot represents the mean DGGR‐lipase activity, and the whisker represents the SD. “n” is the number of dogs evaluated in each re‐visit.

Neither the CPCSS nor DGGR‐lipase activity changed significantly over the follow‐up period (Figures 2 and 3). Overall, there was an association between DGGR‐lipase activity and the CPCSS (*P* = .01). The CPCSS (*P* = .96), or DGGR‐lipase activity (*P* = .55) over time were both not associated with administration of immunosuppressive therapy. For every 1000 U/L increase in DGGR‐lipase activity, the CPSS increases by 0.6 (95% CI, 1.015‐1.123). 1,2‐o‐dilauryl‐rac‐glycero glutaric acid‐(6′‐methylresorufin) ester‐lipase activity was significantly, albeit weakly correlated with the CPSS (*r* = 0.233, *P* = .007). Serum DGGR‐lipase activity poorly discriminated mild‐moderate from severe clinical disease (ROC AUC, 0.64; 95% CI, 0.53‐0.75; *P* = .01). The optimal cutoff point, 428 U/L, corresponded to sensitivity of 65% and specificity of 63%.

Serum CRP concentration (median, 0.0; range, 0.0‐319.1) was measured in 35 visits (26%) and was above RI in 8 visits (23%). Serum CRP was neither correlated with serum DGGR‐lipase activity (*P* = .85) nor with the CPCSS (*P* = .32).

## 4 DISCUSSION

The CPCSS and serum DGGR‐lipase activity were both significantly associated with the type of visit (emergency or elective) to the hospital, which is suggestive of the severity of the disease, thus adding potentially useful information for monitoring CP in dogs. Although serum DGGR‐lipase activity and the CPCSS were significantly associated, the former was poorly predictive of the clinical severity of CP, suggesting that DGGR‐lipase activity has limited utility in monitoring the clinical progression of CP in dogs. Importantly, increases in DGGR‐lipase activity overtime were associated with higher CPCSS, but the impact on the CPCSS was minor, suggesting that only major increases in DGGR‐lipase activity overtime, might be of clinical importance.

The partial clinical utility of DGGR‐lipase activity in monitoring dogs with CP was possibly negatively affected by presence of limited ongoing pancreatic active damage and pancreatic lipase activation and leakage in CP.^15^ While not evaluated in dogs with CP, DGGR‐lipase activity is a sensitive diagnostic marker of AP in dogs. Its specificity is moderate, hampered, among others, by presence of extra‐pancreatic diseases.^9^, ^15^, ^30^, ^31^ Other pancreatic lipase assays have similar diagnostic limitations. For instance, abnormally high Spec cPLI concentration was recorded in various nonpancreatic diseases (eg, intervertebral disc disease,^32^ mitral valve disease,^33^ hyperadrenocorticism,^34^ and CIEs^35^), and in dogs with AP, the Spec cPL diagnostic sensitivity (21%‐91%) and specificity (74%‐100%) vary.^16^, ^36^, ^37^ There is higher serum DGGR‐lipase activity in dogs with various diseases treated in an intensive care unit, where AP was not clinically diagnosed; yet, presence of pancreatopathy could not be excluded.^30^ Nevertheless, according to that study, serum DGGR‐lipase activity is significantly higher in dogs with sonographic evidence of AP (median, 245 U/L; IQR, 74‐1542), compared to dogs where these are absent (median, 83 U/L; IQR, 41‐186 U/L).^30^ Most importantly, serum DGGR‐lipase activity shows poor to moderate sensitivity, but excellent specificity for histologically confirmed CP.^15^ In the current study, severe extra‐pancreatic comorbidities were primarily ruled out, and to increase specificity, a relatively high inclusion cut‐off value of 250 U/L was selected.^15^, ^17^

Similarly to the present cohort, dogs diagnosed with CP are typically middle‐aged to older at diagnosis.^4^, ^5^ Most dogs herein, males and females, were neutered, in agreement with a previous report, where dogs with CP are more likely to be neutered, regardless of sex.^2^ Although toy, terrier, and nonsporting breeds are reportedly more affected by CP,^2^, ^5^ most dogs herein (67%) were of mixed breed, likely reflecting geographical breed prevalence differences.

The chronic, low‐grade gastrointestinal‐related clinical signs characteristically reported in CP^2^ are consistent with the overall low CPCSSs, even upon emergency visits (median, 5; range, 0‐15 out of a possible maximal CPSS of 19), when dogs were assessed to require further diagnostics and in‐hospital emergency care. Additionally, the characteristic waxing and waning clinical presentation of CP^2^ was also evident herein by a lack of change in CPCSS trajectory over time. Clinical deterioration of CP, and A/CP episodes, are presumably explained by the “necrosis‐fibrosis” theory, suggesting that pancreatic fibrosis reduces its distensibility, resulting in duct obstruction, impairing secretion, culminating into acute flare‐ups, and disease progression.^2^ All the dogs in this cohort survived the follow‐up period, during which they showed little and mild clinical signs; however, chronic supportive treatment was commonly required, and revisits were quite frequent, of which 33% were unscheduled emergency visits. Therefore, it seems that CP, as a sole or major disease, was not life‐threatening during the duration of this study. Nevertheless, dogs sustaining CP should be expected to need frequent revisits, warranting appropriate client education regarding the prognosis and treatment, including its cost, and implications on life quality.

Comorbidities, mainly CKD and endocrinopathies, were frequent in our cohort, and such common disorders are probably inevitable in studies of naturally occurring CP.^2^ Dogs with CP are more likely to sustain concurrent DM, and less commonly hyperadrenocorticism and hypothyroidism.^2^, ^3^, ^38^ Although poorly defined, the association between AP or CP and endocrinopathies was described, with undetermined cause and effect.^39^ For example, hyperlipidemia and hypercholesterolemia occur commonly secondary to DM, and are implicated in the pathogenesis of CP, and vice versa, CP might lead to pancreatic islet loss, with consequent DM.^40^ Other comorbidities documented (ie, neoplasia and CKD) likely occurred independently of CP and were age‐related.^41^, ^42^ Yet, CKD and lymphocytic portal hepatitis might have been sequels of systemic or local inflammation (respectively), or were possibly secondary complications of A/CP (eg, dehydration affecting kidney perfusion).^4^, ^10^, ^41^, ^43^

Immune‐mediated CP is reported in cocker spaniel and Cavalier King Charles spaniel breeds in the United Kingdom^5^, ^44^ and administrating glucocorticoids or cyclosporine is reported in dogs and cats with CP and AP to suppress inflammation.^45^, ^46^ Immunosuppressive therapy was not associated with the clinical score or with serum DGGR‐lipase activity changes (either improved or worsened) herein; nevertheless, these results should be interpreted cautiously. Only 1 spaniel dog was included in the present study, suggesting that immune‐mediated CP was possibly uncommon in this cohort.^5^, ^44^

C‐reactive protein is a useful marker for assessing severity and prognosis of AP in dogs, although not an early disease marker, since its maximal concentrations are documented days from disease onset.^14^, ^21^ In the current study, serum CRP concentration was not associated with neither the CPCSS nor with serum DGGR‐lipase activity, suggesting that CRP is less useful for monitoring clinically milder, or localized, chronic pancreatic disease. Nevertheless, CRP was measured in only 26% of the revisits, warranting future larger‐scale studies.

This study has several limitations, mainly attributed to its retrospective nature, including variable monitoring and treatment protocols, implemented by different clinicians, and the variable number of revisits per dog. While collection of clinical score indices was performed by a single investigator, some potential inaccuracies might have occurred when retrospectively and subjectively assessing the attitude and appetite of dogs upon visits. Furthermore, while some acute changes are reflected by the CPCSS, some of the clinical indices reflect changes that have occurred over a longer time period. The latter might have contributed to the weak correlation with serum DGGR‐lipase activity, which is a single‐point measure of a leakage enzyme, reflecting active pancreatic tissue damage. A shorter duration of clinical signs before presentation is associated with higher DGGR‐lipase activities in AP, affecting its diagnostic performance.^30^ In CP, it is plausible that variable intervals between DGGR‐lipase activity measurements, as well as acute flare‐ups, or presence of active pancreatic inflammation, affect the diagnostic accuracy of DGGR‐lipase activity in reflecting clinical severity. Nonetheless, the visits herein were frequent (median between‐visit time interval, 17 days), and even small changes (eg, <5%) in body weight affected the clinical score; thus, the CPCSS did reflect recent changes. Small changes in the score of individual clinical signs, or concurrent small score changes in several clinical signs, might lead to an overall change in the severity of clinical CP. Thus, cautious interpretation is recommended in actual clinical settings, as such changes do not necessarily justify therapeutic or monitoring changes. Additionally, while efforts were made to rule out comorbidities that potentially affected the CPCSS score, possibly, concurrent diseases were undiagnosed in some dogs. On the other hand, the rather strict inclusion criteria potentially led to loss of dogs with CP, which if included, would have increased the size of this limited cohort, possibly strengthening the statistical analyses. Treading this fine line between 2 conflicting trends was deemed necessary for obtaining reliable results. Still, some comorbidities and treatments might have affected DGGR‐lipase activity. Specifically, several dogs in the current study were exposed to either exogenous or increased endogenous glucocorticoids.^34^, ^47^ Yet, in a study evaluating prednisolone effect on DGGR‐lipase activity, the latter was only mildly increased (from activity range between 14.45‐31.48 U/L and 15.91‐48.48 U/L), and all results were within RI.^48^ Additionally, the higher DGGR‐lipase activity in dogs with hyperadrenocorticism is mostly mild (median, 180 U/L),^49^ and lower than the currently set cutoff of >250 U/L. Thus, higher DGGR‐lipase activity in hypercortisolemic dogs most likely reflects ongoing CP rather than merely the mild sole glucocorticoid effect on pancreatic lipase level.^50^ Decreased glomerular filtration rate, resulting in decreased DGGR‐lipase clearance might have also contributed to increase in its activity.^51^ Nevertheless, experimental and clinical studies of dogs with kidney injury support the presence of ongoing pancreatic damage as a more probable source of higher DGGR‐lipase activities.^30^, ^50^, ^51^ In this cohort, a minority of dogs had low stage and stable CKD, and it is therefore likely that CKD had marginal effect on DGGR‐lipase activity. Another common limitation to many studies of pancreatitis in dogs is the lack of pancreatic histopathology.^2^, ^16^ Thus, CP was not definitely diagnosed, and pancreatic inflammation and structural changes were not examined. Therefore, possibly dogs with recurrent AP, mimicking ongoing CP, were included.^2^ Nevertheless, this distinction between recurrent AP and “true” CP is less important in terms of clinical management, as their treatment and complications (eg, DM)^2^ are virtually the same.^2^, ^52^ Finally, the study was conducted in a tertiary‐care hospital, which likely introduced selection bias toward complicated and refractory CP cases, and highly compliant dog owners, which might have affected results. Nevertheless, the monitoring measures assessed in this study are widely accessible in primary care clinic setup, providing useful data regarding interpretation and limitations of serum DGGR‐lipase activity in dogs with CP.

In conclusion, serum DGGR‐lipase activity is only weakly correlated with the CPCS in dogs with CP.

## CONFLICT OF INTEREST DECLARATION

Authors declare no conflict of interest.

## OFF‐LABEL ANTIMICROBIAL DECLARATION

Authors declare no off‐label use of antimicrobials.

## INSTITUTIONAL ANIMAL CARE AND USE COMMITTEE (IACUC) OR OTHER APPROVAL DECLARATION

This is a retrospective study, based on data collected from medical files. An ethical approval was not required.

## HUMAN ETHICS APPROVAL DECLARATION

Authors declare human ethics approval was not needed for this study.

## Supporting information


**Data S1.** Supporting Information.

**Table S1.** Complete blood count results upon diagnosis of chronic pancreatitis in 24 dogs presented to the hospital.
**Table S2.** Serum chemistry results upon diagnosis of chronic pancreatitis in 24 dogs presented to the hospital.

**Table S3.** Associations between DGGR‐lipase activity and presence of clinical signs that constitute the Canine Chronic Pancreatitis Clinical Severity scoring system in 134 visits to the hospital of 24 dogs with chronic pancreatitis.
