# Supplementary material for: Utility of 1,2‐o‐dilauryl‐rac‐glycero glutaric acid‐(6′‐methylresorufin)‐ester‐lipase for monitoring dogs with chronic pancreatitis
Source: J Vet Intern Med. 2023 Feb 13;37(2):437–45. doi: 10.1111/jvim.16638 (PMC10061187; doi:10.1111/jvim.16638)
Supplement: Supplementary file 2 — Table S1. Complete blood count results upon diagnosis of chronic pancreatitis in 24 dogs presented to the hospital. Table S2. Serum chemistry results upon diagnosis of chronic pancreatitis in 24 dogs presented to the hospital. [file JVIM-37-437-s001.pdf]

Supplementary Table 1: Complete blood count results upon diagnosis of chronic pancreatitis in 24 dogs presented to the hospital.

Analyte	Median (range) (n = 24)	Within RI n (%)	n (%) >RI	n (%) <RI	RI
Leukocytes (x10^3/mm^3)	9.3 (4.9-19.1)	20 (83)	3 (13)	1 (4)	5.2-13.9
Neutrophils (x10^3/mm^3)	6.5 (3.0-16.5)	18 (75)	5 (21)	1 (4)	3.9-8.0
Lymphocytes (x10^3/mm^3)	1.65 (0.6-4.1)	15 (63)	0 (0)	9 (37)	1.3-4.1
Monocytes (x10^3/mm^3)	0.52 (0.22-1.63)	22 (92)	2 (8)	0 (0)	0.2-1.1
Eosinophils (x10^3/mm^3)	0.27 (0.02-1.5)	21 (88)	3 (12)	0 (0)	0.0-0.6
Basophils (x10^3/mm^3)	0.02 (0.0-0.1)	24 (100)	0 (0)	0 (0)	0.0-0.1
LUC (x10^3/mm^3)	0.03 (0.0-0.75)	23 (96)	1 (4)	0 (0)	0.0-0.3
Red blood cells (x10^6/mm^3)	6.0 (4.9-7.8)	16 (67)	0 (0)	8 (33)	5.7-8.8
Hemoglobin (g/dL)	13.7 (11.0-18.0)	17 (71)	0 (0)	7 (29)	12.9-18.4
Hematocrit (%)	39.7 (33.7-53.9)	19 (79)	0 (0)	5 (21)	37.1-57.0
MCV (fL)	68.4 (61.5-76.6)	20 (83)	4 (17)	0 (0)	58.8-71.2
MCHC (g/dL)	33.5 (31.6-37.0)	23 (96)	1 (4	0 (0)	31.0-36.2
RDW (%)	13.5 (11.2-31.1)	16 (67)	8 (33)	0 (0)	11.9-14.5
Platelets (x10^3/mm^3)	398 (200-646)	13 (54)	11 (46)	0 (0)	143.3-400.0
MPV (fL)	11.4 (8.1-16.9)	12 (50)	12 (50)	0 (0)	7.0-11.0
PCT (%)	0.48 (0.26-0.73)	7 (29)	17 (71)	0 (0)	0.1-0.4
PDW (%)	56.4 (48.6-68.5)	22 (92)	2 (8)	0 (0)	40.6-65.2

RI, reference interval; LUC, large unstained cells; MCV, mean corpuscular volume; MCHC, mean corpuscular hemoglobin concentration; RDW, red blood cell distribution width; PCT, platelet crit; PDW, platelet distribution width; MPV, mean platelet volume.

Supplementary Table 2: Serum chemistry results upon diagnosis of chronic pancreatitis in 24 dogs presented to the hospital.

Analyte	Median (range) (n = 24)	Within RI n (%)	> RI n (%)	< RI n (%)	Reference interval
Alanine transaminase (U/L)	72.5 (27-222)	10 (42)	14 (58)	0 (0)	19-67
Albumin (g/dL)	3.85 (3.0-4.9)	23 (96)	1 (4)	0 (0)	3.0-4.4
Alkaline phosphatase (U/L)	247 (22-2362)	10 (42)	14 (58)	0 (0)	21-170
Amylase (U/L)	1083 (278-4891)	20 (83)	4 (17)	0 (0)	103-1510
Aspartate transaminase (U/L)	28.5 (13-65)	17 (71)	5 (21)	2 (8)	19-42
Bilirubin (mg/dL)	0.15 (0.1-0.17)	24 (100)	0 (0)	0 (0)	0.0-0.2
Chloride (mmol/L)	107 (96-117)	16 (67)	0 (0)	8 (33)	104-118
Cholesterol (mg/dL)	276 (155-743)	17 (71)	7 (29)	0 (0)	135-361
Creatine kinase (U/L)	124 (48-286)	23 (96)	0 (0)	1 (4)	51-399
Creatinine (mg/dL)	0.9 (0.5-2.0)	16 (67)	8 (33)	0 (0)	0.3-1.2
γ-glutamyl transpeptidase (U/L)	4.0 (0.0-48.0)	19 (79)	5 (21)	0 (0)	0.0-6.0
Globulin (g/dL)	2.6 (2.1-4.4)	23 (96)	1 (4)	0 (0)	1.8-39
Glucose (mg/dL)	106 (85-670)	19 (79)	5 (21)	0 (0)	64-123
Phosphorus (mg/dL)	4.0 (2.9-4.9)	23 (96)	0 (0)	1 (4)	3.0-6.2
Potassium (mmol/L)	4.8 (3.8-5.35)	22 (92)	0 (0)	2 (8)	3.6-5.3
Sodium (mmol/L)	145 (135-152)	21 (88)	0 (0)	3 (12)	140-154
Total calcium (mg/dL)	10.0 (9.5-11.5)	20 (83)	0 (0)	4 (17)	9.7-11.5
Total protein (g/dL)	6.45 (5.6-8.0)	23 (96)	1 (4)	0 (0)	5.4-7.6
Triglycerides (mg/dL)	86 (34-1193)	16 (67)	8 (33)	0 (0)	19-133
Urea (mg/dL)	43 (11-86)	16 (67)	8 (33)	0 (0)	10.7-53.5

RI, reference interval.
