# Supplementary material for: Utility of 1,2‐o‐dilauryl‐rac‐glycero glutaric acid‐(6′‐methylresorufin)‐ester‐lipase for monitoring dogs with chronic pancreatitis
Source: J Vet Intern Med. 2023 Feb 13;37(2):437–45. doi: 10.1111/jvim.16638 (PMC10061187; doi:10.1111/jvim.16638)
Supplement: Supplementary file 3 — Table S3. Associations between DGGR‐lipase activity and presence of clinical signs that constitute the Canine Chronic Pancreatitis Clinical Severity scoring system in 134 visits to the hospital of 24 dogs with chronic pancreatitis. [file JVIM-37-437-s002.pdf]

Supplementary Table 3: Associations between DGGR-lipase activity and presence of clinical signs that constitute the Canine Chronic Pancreatitis Clinical Severity scoring system in 134 visits to the hospital of 24 dogs with chronic pancreatitis.

Clinical sign	Clinical sign absent* DGGR-lipase activity (U/L) Mean (95%CI)	Clinical sign present** DGGR-lipase activity (U/L) Mean (95%CI)	*P* value
Attitude	524 (416-631)	1123, 297.1	0.03
Appetite	611 (267-590)	828 (579-1077)	0.25
Vomiting	646 (347-945)	900 (499-1300)	0.32
Stool consistency	678 (451-904)	817 (331-1302)	0.51
Stool frequency	725 (462-987)	327 (249-405)	0.01
Weight loss	772 (462-1081)	622 (392-852)	0.23
Abdominal pain	617 (445-789)	1031 (295-1768)	0.25

The associations between DGGR-lipase activity and presence of each clinical sign were examined using Generalized Estimating Equations. Alpha was set at 0.05; 95%CI, 95% confidence interval; *, score 0; **, scores ranging between from 1-3 for changes in attitude, appetite, vomiting, school consistency, stool frequency and weight loss, and score 1 for presence of abdominal pain.
